# Supplementary material for: Simultaneously achieving giant piezoelectricity and record coercive field enhancement in relaxor-based ferroelectric crystals
Source: Nat Commun. 2022 May 4;13:2444. doi: 10.1038/s41467-022-29962-6 (PMC9068613; doi:10.1038/s41467-022-29962-6)
Supplement: Supplementary file 1 — Supplementary Information [file 41467_2022_29962_MOESM1_ESM.pdf]

Supplementary Information

Simultaneously achieving giant piezoelectricity and record coercive field enhancement in relaxor-based ferroelectric crystals

Liya Yang,#[1,2,3] Houbing Huang,#[4] Zengzhe Xi,[5] Limei Zheng,[1,*] Shiqi Xu,[4] Gang Tian,[1] Yuzhi Zhai,[1] Feifei Guo,[5] Lingping Kong,[6] Yonggang Wang,[6] Weiming Lü,[7,*] Long Yuan,[8] Minglei Zhao,[1] Haiwu Zheng,[2] and Gang Liu[6,*]

[1]School of Physics, State Key Laboratory of Crystal Materials, Shandong University, Jinan 250100, China

[2]International Joint Research Laboratory of New Energy Materials and Devices of Henan Province, School of Physics and Electronics, Henan University, Kaifeng 475004, China

[3]Condensed Matter Science and Technology Institute, School of Instrumentation Science and Engineering, Harbin Institute of Technology, Harbin 150080, China

[4]School of Materials Science and Engineering & Advanced Research Institute of Multidisciplinary Science, Beijing Institute of Technology, Beijing 100081, China

[5]School of Materials and Chemical Engineering, Xi'an Technological University, Xi'an 710032, China

[6]Center for High Pressure Science and Technology Advanced Research, Shanghai 201203, China

[7]Spintronics Institute, School of Physics and Technology, University of Jinan, Jinan 250022, China

[8] Key Laboratory of Functional Materials Physics and Chemistry of the Ministry of Education, Jilin Normal University, Changchun 130103, China

*Corresponding authors:

zhenglm@sdu.edu.cn (Limei Zheng);

sdy.lvwm@ujn.edu.cn (Weiming Lü);

liugang@hpstar.ac.cn (Gang Liu)

Note 1. Lattice anisotropy of $Pb(Sc_{1/2}Nb_{1/2})O_3$ system

The lattice parameters of $Pb(Sc_{1/2}Nb_{1/2})O_3$ (PSN) and $Pb(Mg_{1/3}Nb_{2/3})O_3$ (PMN) are listed in Table S1, where the shear lattice deformation is estimated by (90°-α)/2. Both materials are rhombohedral (R) structures at room temperature. In comparison with PMN (ICSD #161663), PSN (ICSD #90501) has larger lattice parameters, and exhibits ~3 times larger of the shear deformation. Thus, the introduction of PSN into Pb-based ferroelectrics results in a high level of lattice anisotropy. For instance, the lattice deformation (estimated by c/a-1) of PSN-0.42PT are ~5 times higher than that of PMN-0.32PT (both compounds are with MPB composition). As such, we may consider introduce PSN into the PMN-PT system to establish local structure heterogeneity with strong tetragonality and large lattice deformation c/a-1.

Table S1. Lattice parameters and lattice deformation of various PSN- and PMN-based perovskites.

	Phase	Lattice parameter	Lattice deformation	Deformation Ratio [a]
PSN	Rhombohedral	a=b=c=4.082 Å<br>α=β=γ=89.914 ^{o}	(90°-α) /2=0.043^o	3
PMN	Rhombohedral	a=b=c=4.045 Å<br>α=β=γ=89.971 ^{o}	(90°-α) /2=0.015^o	
PSN-0.42PT[1]	Monoclinic *Pm*	a=4.036 Å<br>b=3.987 Å<br>c=4.091 Å<br>β=90.190 ^{o}	c/a-1=1.36%	6
PMN-0.32PT[2]	Monoclinic *Pm*	a=4.018 Å<br>b=4.005 Å<br>c=4.028 Å<br>β=90.146 ^{o}	c/a-1=0.23%	

[a] Deformation ratio is the ratio of lattice deformation of PSN system to PMN system. For the pure PSN and PMN, shear deformation is adopted; for the binary PSN-PT and PMN-PT, normal lattice deformation is considered.

Note 2. Repeatability and frequency dependence of coercive field

To verify the repeatability of the extraordinary large coercive field E_C, we measured P-E hysteresis loops of three different $[001]_C$ oriented 0.06PSN-0.61PMN-0.33PT crystals. All samples exhibit similar E_C values, ~8.0 kV/cm (Figure S1), indicating that the large coercive field is an intrinsic behavior rather than accidental phenomena.

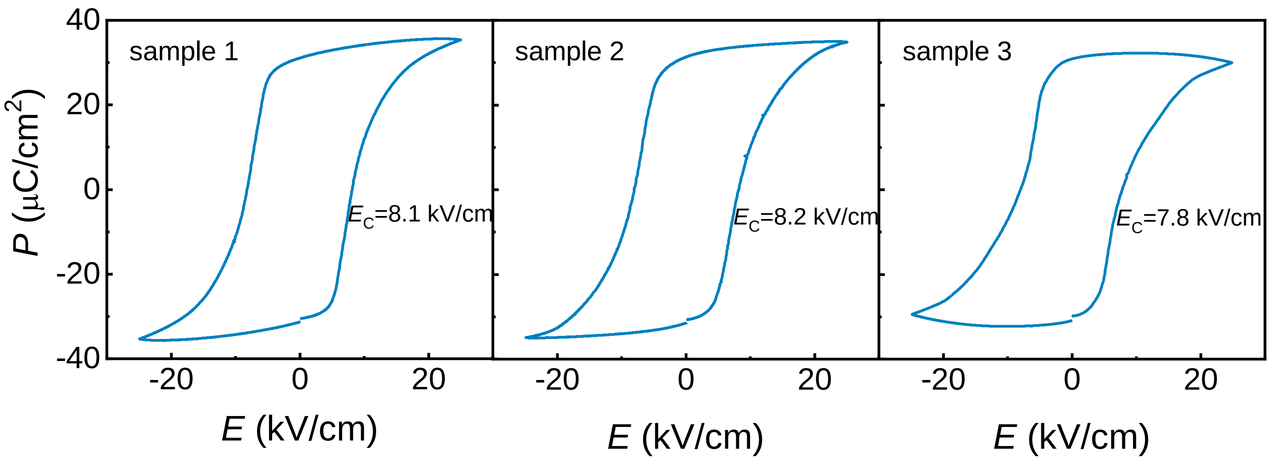

Figure S1. Repeatability of the extraordinary large coercive field convinced in three different 0.06PSN-0.61PMN-0.33PT samples, showing E_C values of 8.1, 8.2, and 7.8 kV/cm, respectively.

Figure S2a shows the P-E hysteresis loops of 0.06PSN-0.61PMN-0.33PT at the frequency $0.1 \le f \le 100$ Hz. The frequency dependence of E_C was summarized in Figure S2b, from which one can see E_C increases from 7.5 kV/cm to 11.8 kV/cm as f increases from 0.1 Hz to 100 Hz. The frequency dependent E_C can be described by the theoretical model from Ishibashi and Orihara [3,4]

$$E_C = Kf^{\beta} . \tag{S1}$$

And our experimental data in Figure S2b can be well described by Equation (S1) with derived parameters of K=2.51 and β=0.07. According to relationship, the coercive field at other frequencies can be estimated.

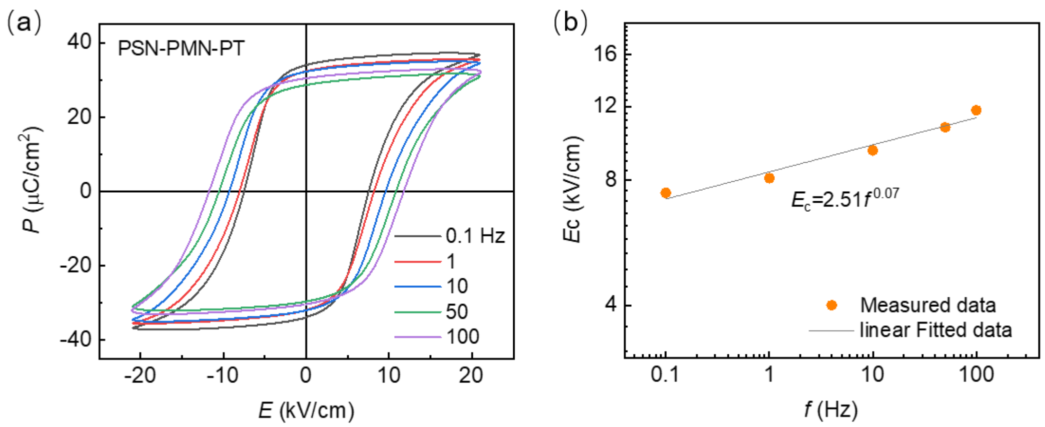

Figure S2. Frequency dependence of P-E hysteresis loops (a) and E_C (b) for 0.06PSN-0.61PMN-0.33PT single crystal. Fitting the experimental data by Eq. S1 gives $E_C=2.51f^{0.07}$.

Note 3. Current-electric field (*I*-*E*) loops

Figure S3a shows the *I*-*E* loops of the crystal sample (used in Figure S2) measured at different frequencies. For each frequency, a current peak can be observed around E_C, corresponding to the displacement current originated from domain switching. The maximum displacement current increases with frequency for the shortened switching time. Figure S3b summarizes *I*-*E* loops of 4 different samples measured at a fixed frequency of 1 Hz (Samples 1-3 correspond to the 3 samples in Supplementary Figure S1). For all the samples, the conductive current is negligible in comparison with the displacement current. The enlarged *I*-*E* curves are provided as the inset of Figure S3b. Only Sample 3 demonstrates a detectable leakage current while other samples show excellent insulation effect.

Note 4. Temperature dependence of material properties of PSN-PMN-PT

Figure S4 shows the normalized temperature dependence of d_{33}, d_{15}, k_{33} and k_{15} of 0.06PSN-0.61PMN-0.33PT single crystals below $T_{F\text{-}F}$. k_{33} and k_{15} is almost temperature independent with variation below 3% and 13%, respectively, manifesting excellent thermal stability. Shear piezoelectric coefficient d_{15} increases slight by 30%, while d_{33} increases dramatically by 140% as approaching phase transition temperature $T_{F\text{-}F}$.

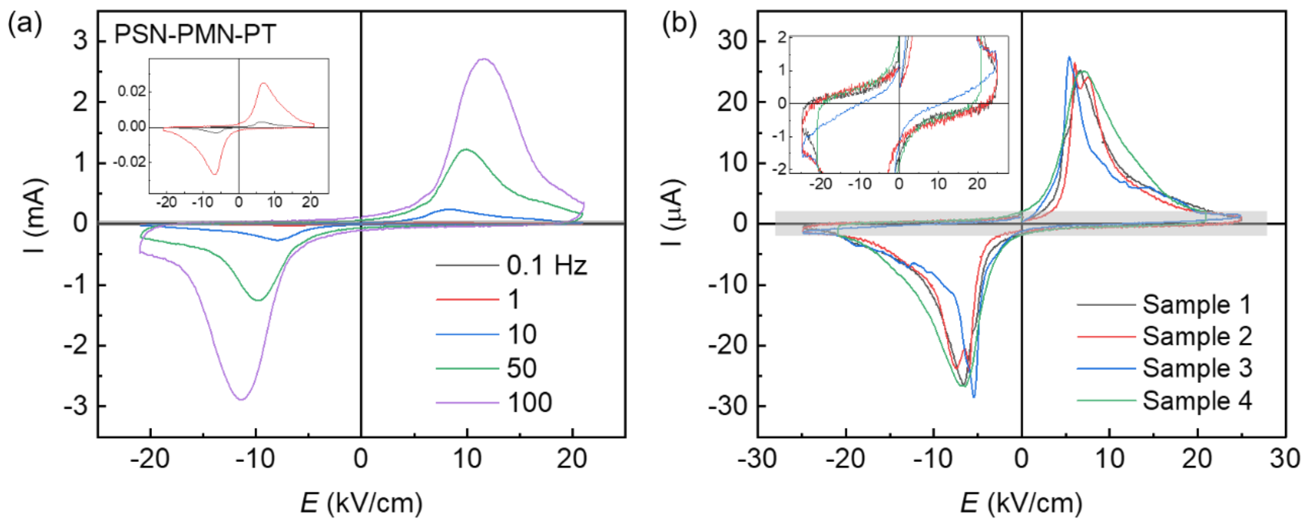

Figure S3. *I*-*E* loops of the 0.06PSN-0.61PMN-0.33PT single crystals. (a) Frequency dependence of *I*-*E* loops for Sample 4. (b) *I*-*E* loops of 4 different samples measured at 1 Hz.

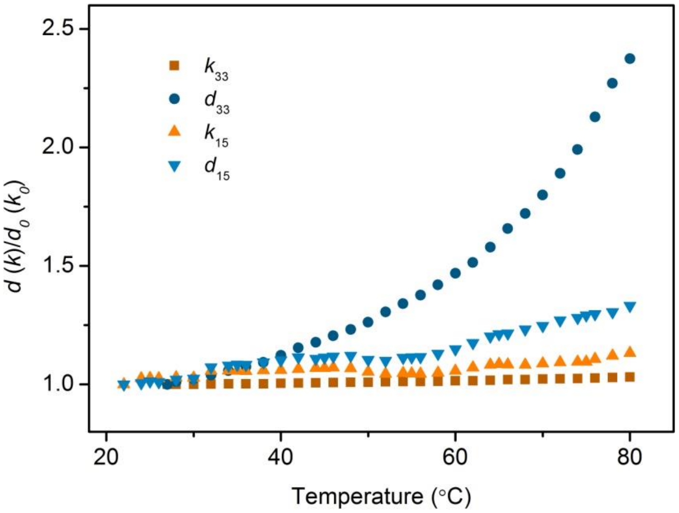

Figure S4. Normalized d_{33}, d_{15}, k_{33} and k_{15} as a function of temperature.

Figure S5a shows the temperature dependent *P-E* hysteresis loops for 0.06PSN-0.61PMN-0.33PT single crystal. The maximum polarization P_{max}, remnant polarization P_r, and the coercive field E_C as a function of temperature are summarized in Figure S5b. E_C maintains a high value above 6 kV/cm till temperature increases to $T_{F\text{-}F}$. Below 150 °C, well-saturated *P-E* loops can be obtained, from which one can see P_{max} and P_r decrease gradually with temperature. As approaching Curie temperature, the *P-E* loop shrinks obviously and P_r reduces dramatically. High polarization values with P_{max}=12 μC/cm^2 and P_r=3.5 μC/cm^2 can even be achieved at 210°C, 50°C above Curie temperature T_C.

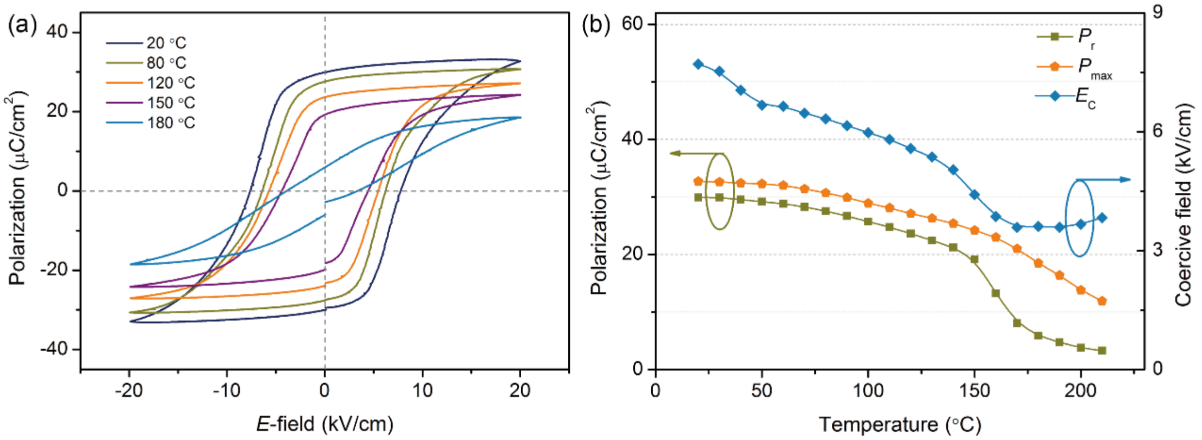

Figure S5. Temperature dependence of ferroelectric properties for 0.06PSN-0.61PMN-0.33PT single crystal. (**a**) *P-E* hysteresis loops and (**b**) remnant polarization P_r, maximum polarization P_{max} and E_C as a function of temperature. All the loops are measured at 1 kHz. E_C above 6.2 kV/cm is maintained below $T_{F\text{-}F}$.

Note 5. Domain switching and local piezoelectric response of PSN-PMN-PT

We carried out piezoelectric force microscopy (PFM) to *in situ* map the ferroelectric domain patterns and investigate the local piezoelectric response. We employed a gradually increasing tip-voltage V_{dc} from 0 to 10 V on the crystal surface, enabling us to pole a local area of 3×3 μm^2 in a smooth manner. Figure S6a shows the evolutions of the out-of-plane PFM amplitude and phase images, from which the amplitude and phase response along the white dash line were derived and quantitatively analyzed (Figure S6b). The 180° phase contrast between the recording area measured at 5 V and 8 V reveals the complete polarization reversal of the ferroelectric domains, which is further supported by switching spectroscopy PFM (SS-PFM) characterization also displaying a 180° phase contrast (Figure S6c). According to the fact that the enhanced domain switching and mobility contribute significantly to the giant piezoelectric response, such a fully polarization switch is critically necessary for high performance of ferroelectrics, especially for materials with a large E_C. As shown in Figure S6d, the PSN-PMN-0.33PT crystal features a well-defined local piezoelectric response loop, presenting direct evidence for a superior piezoelectric response from a microscopic perspective. Since the vertical vibration signal is directly related to the piezoelectric response, the macroscopic piezoelectric property can be viewed as a collective effect of the microscopic piezoelectric response.

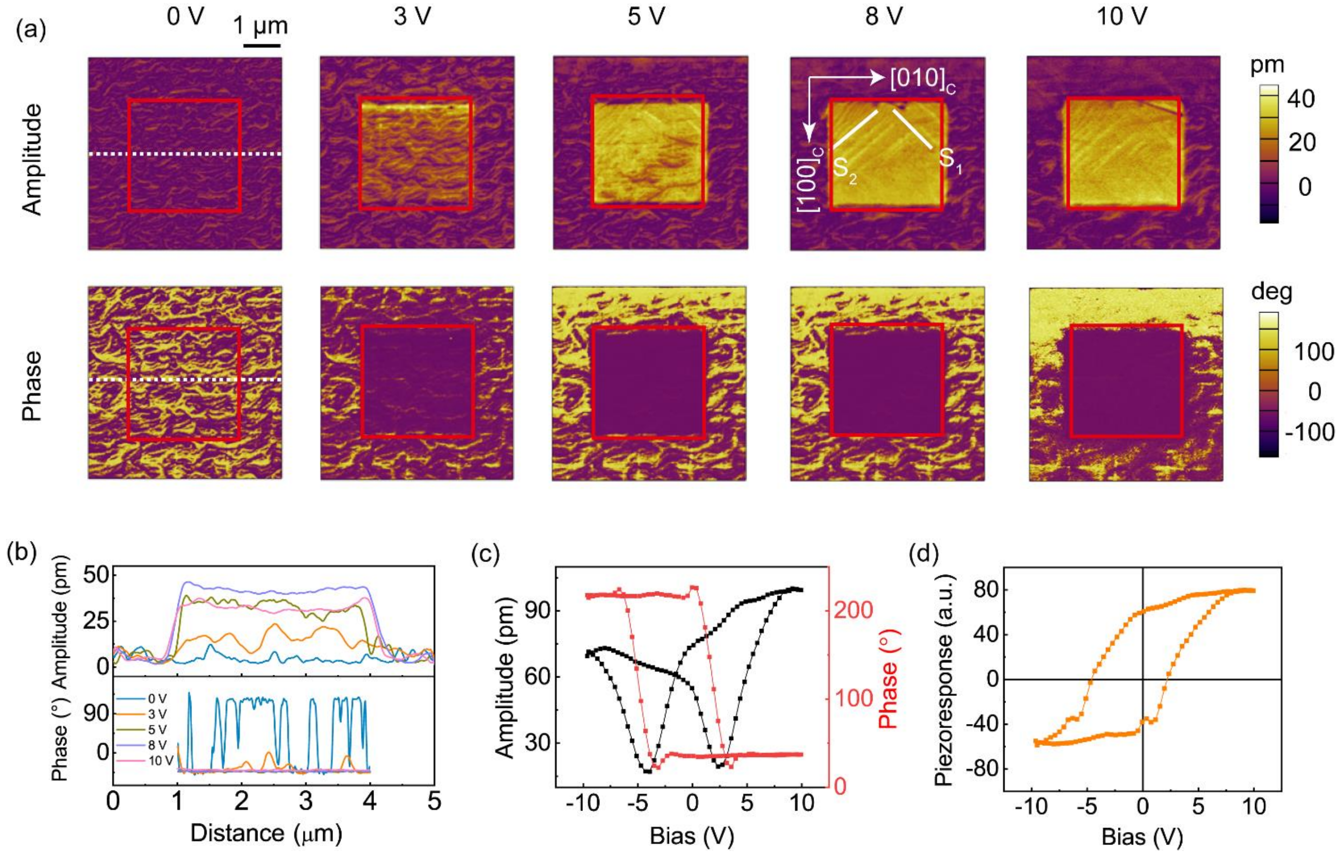

Figure S6. Domain structures and local domain dynamics measured by PFM. (**a**) Evolution of vertical PFM amplitude and phase structures under different tip voltage V_p. The amplitude and phase response along the white dash are extracted and shown in (**b**). (**c**) Local amplitude and phase and (**d**) piezoresponse as a function of tip voltage measured by SS-PFM. The local piezoresponse is calculated by $PR=A\times\cos(\varphi)$, where PR, A, and φ indicate local piezoresponse, amplitude, and phase angle, respectively.

Figures S7a-l show the domain evolution with temperature for 0.06PSN-0.61PMN-0.33PT single crystals. The homogeneous labyrinth-like nanodomain structures show slight changes below $T_{F\text{-}F}$. Above $T_{F\text{-}F}$, however, the labyrinth domains expand dramatically with temperature, and the stripe-like domains with domain walls along $[010]_C$ direction appears, demonstrating that the crystal changes from MPB to T phase. As temperature further approaches T_C, both amplitude and phase contrast between different nano-domains attenuated, corresponding to degradation of ferroelectric domain structure.

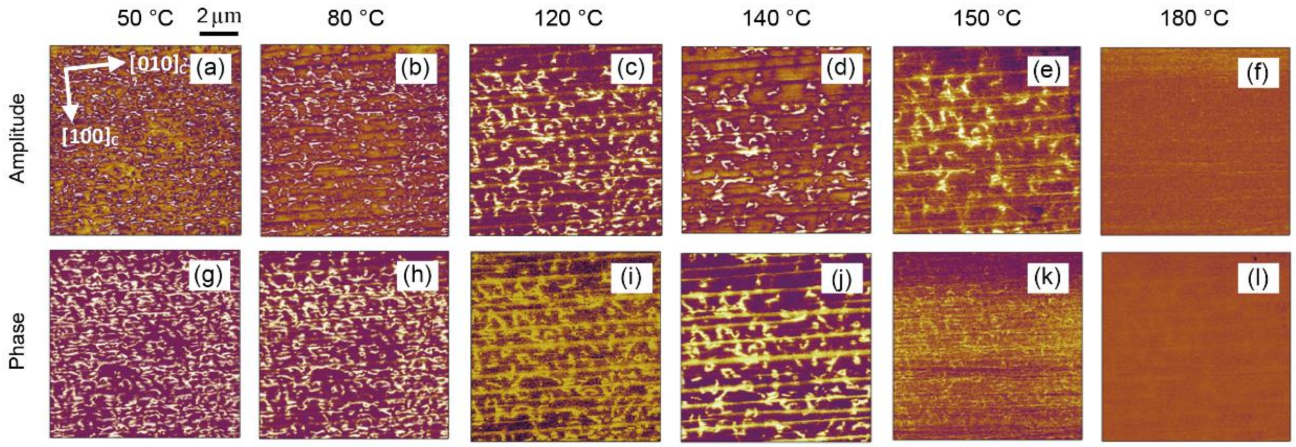

Figure S7. Domain evolution with temperature for 0.06PSN-0.61PMN-0.33PT single crystals. (**a-l**) The PFM amplitude and phase images at various temperatures.

Note 6. Autocorrelation function of the polar regions.

Figure S8a shows the PFM images of $[001]_C$ oriented 0.06PSN-0.61PMN-0.33PT and 0.67PMN-0.33PT crystals. Ternary 0.06PSN-0.61PMN-0.31PT consists of smaller domains than the binary system (~200 nm for PSN-PMN-PT *vs*. ~1300 nm for PMN-PT). We further quantify the agglomerates of the local polar regions by an averaged autocorrelation function over all in-plane directions as: [5-7]

$$\langle C(r) \rangle = \sigma^2 \exp\left[-\left(\frac{r}{\xi} \right)^{2h} \right]. \qquad (1)$$

Here σ is a constant and ξ is short-range correlation length that indicates the degree of polarization correlation, and h ($0<h<1$) describes the roughness of the polarization interface, and the fractal dimension of an interface is d=3-h. The best fitting results are shown in Figure S8b. ξ=70 nm is obtained in 0.06PSN-0.61PMN-0.33PT crystal, much smaller than those from analysis on 0.67PMN-0.33PT (ξ=450 nm), demonstrating it is much more difficult for the ternary crystal to establish homogeneous polarization order but only short-range order between neighboring clusters, echoing to the stronger relaxor behavior shown in Figures 3b and 3c.

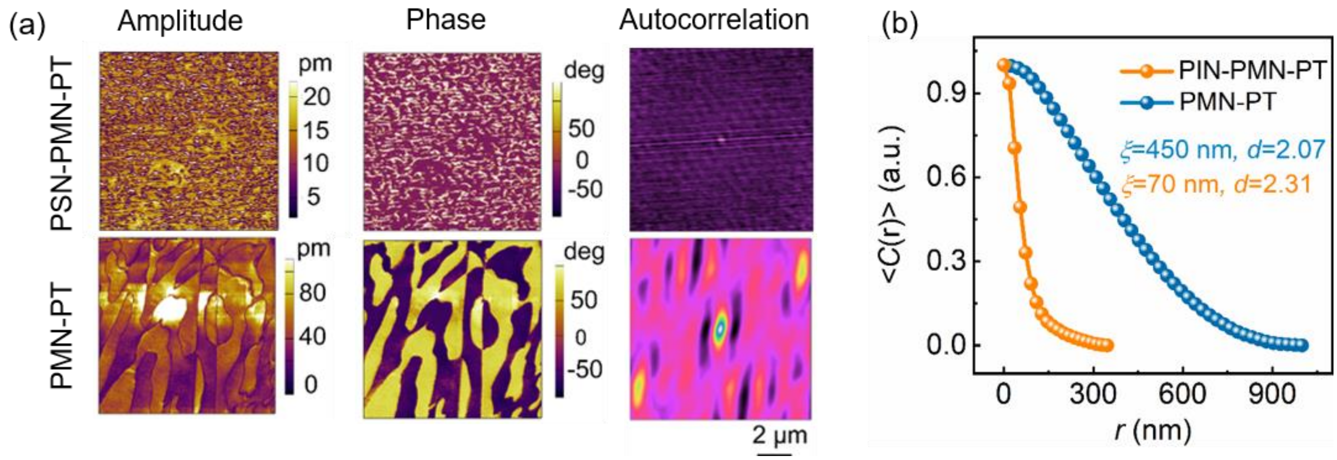

Figure S8. (**a**) Amplitude, phase and autocorrelation images and (**b**) the averaged autocorrelation function for the two crystals and the best fitting of the experimental points.

Figures S9a and b shows the temperature dependent autocorrelation analysis, and in Figure S9c and d we extracted the temperature dependent correlation radius ξ and fractal dimension d, respectively. Initially, both ξ and d change slightly with temperature, demonstrating a stable domain structure and random fields with temperature. As approaching freezing temperature T_{VF}, ξ increases by 2 times (from 85 nm to 171 nm) and d decreases dramatically, indicating that the local structure becomes more ordered. Above T_{VF}, some of the static PNRs transform into dynamic ones, the system becomes more disordered, resulting in the decrease of ξ and increase of d. Nevertheless, at 180 ^{o}C, 25 ^{o}C above T_m, ξ (~30 nm) is still not negligible, denoting the existence of quasi-static PNRs. With temperature further increasing above 180 ^{o}C, the quasi-static PNRs are not detectable, only dynamic PNRs exist.

Note 7. Temperature dependence of dielectric constant for 0.67PMN-0.33PT single crystal.

Figure S10 shows the temperature dependent dielectric constant around T_m for the unpoled 0.67PMN-0.33PT single crystals, from which Vogel-Fulcher relationship can be fitted. In addition, we observed a 2.5 K of T_m shift, which is much smaller than that of ternary crystals with same PT content.

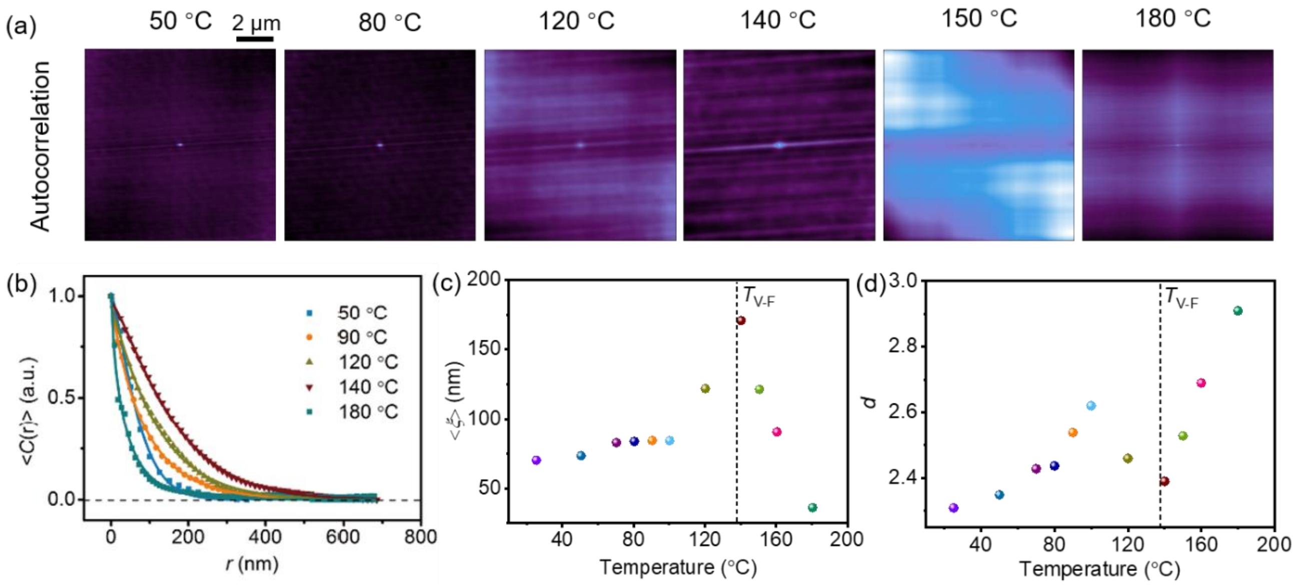

Figure S9. Temperature dependent autocorrelation analysis. (a) The autocorrelation images at various temperatures. (b) The temperature evolution of the averaged autocorrelation function and fitting results. (c) and (d) average autocorrelation length ξ and fractional dimension d as a function of temperature, respectively.

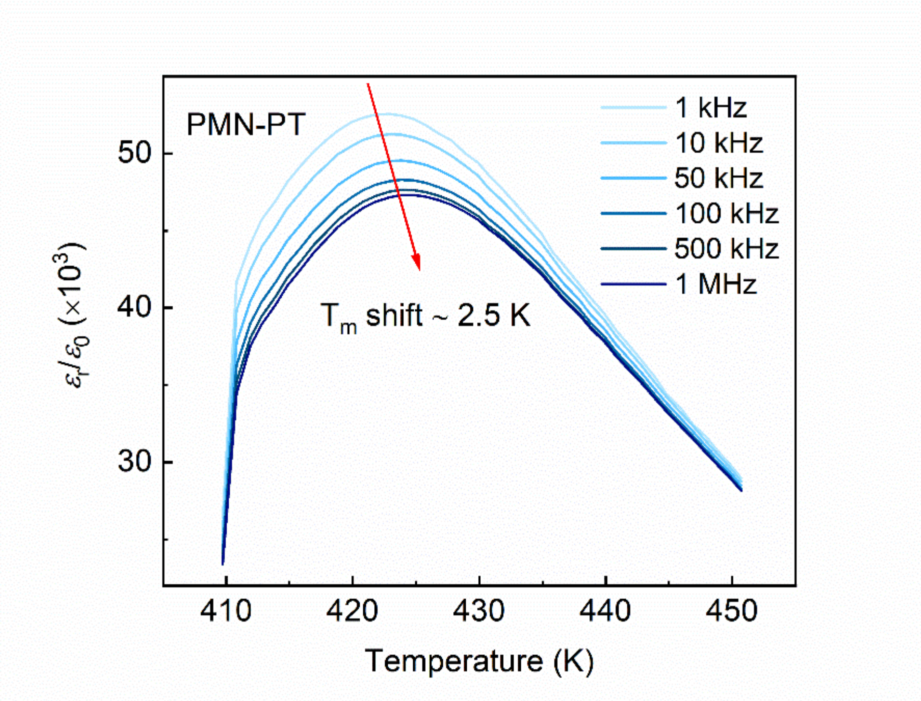

Figure S10. High-temperature dielectric property for unpoled 0.67PMN-0.33PT single crystal.

Note 8. Local structure heterogeneity.

Figures S11a-c and S11e-g represent the selected area electron diffraction (SAED) patterns along $[001]_C$, $[1\bar{2}0]_C$, and $[1\bar{1}3]_C$ axis for 0.67PMN-0.33PT and 0.06PSN-0.61PMN-0.33PT, respectively. These two crystals exhibit similar crystalline structure. Figures S11d and S11h are the dark-field TEM images of the two crystals, respectively, where the long range order ferroelectric domain structures cannot be observed, in accordance with the results observed by PFM (Figure S8). Moreover, the PSN-PMN-PT system exhibits a more complicated local structure heterogeneity.

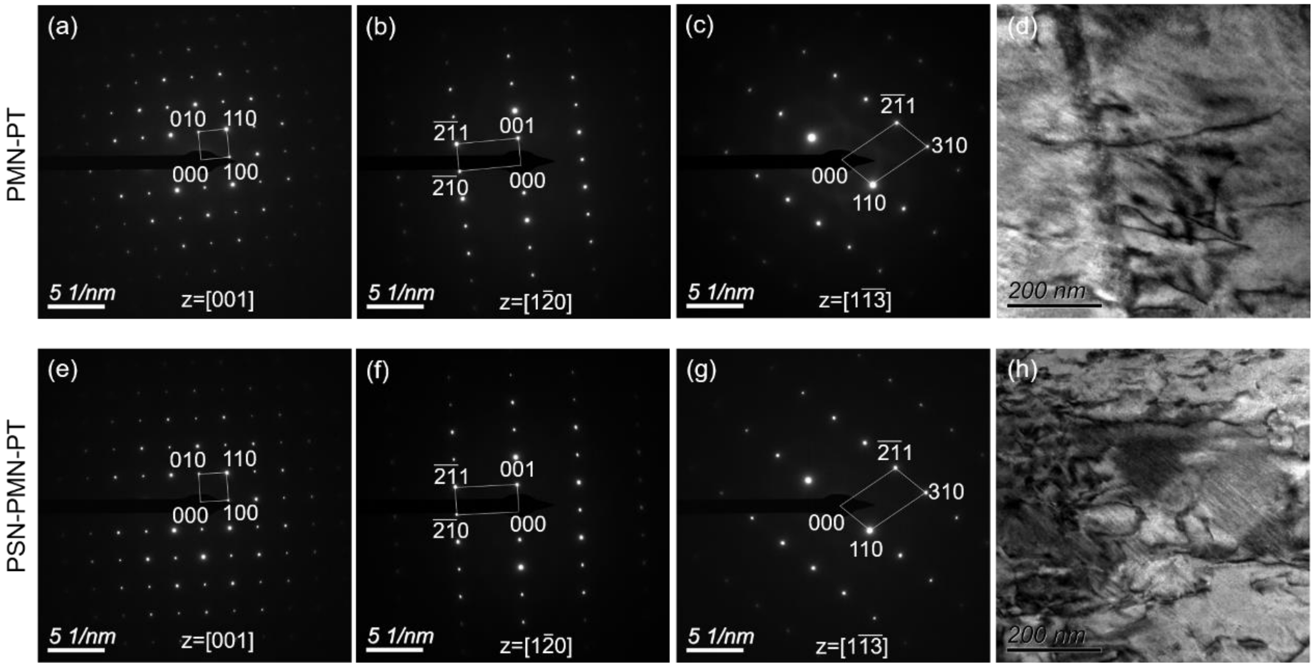

Figure S11. Selected area electron diffraction (SAED) patterns of 0.06PSN-0.61PMN-0.33PT and 0.67PMN-0.33PT single crystals. (**a-c**) are the images along $[001]_C$, $[1\bar{2}0]_C$, and $[1\bar{1}\bar{3}]_C$ axis for PMN-PT, and (**e-g**) are those for PSN-PMN-PT. (**d**) and (**h**) is the local microstructures observed by HRTEM.

Figure S12 is the HAADF-STEM image of 0.67PMN-0.33PT crystal, from which the polarization vector P_S of each unit cell column was determined. Arising from its MPB composition, 0.67PMN-0.33PT also exhibits a multiphase coexistence, *e.g.*, O, T, and M phases, yet the polar regions are larger than that in 0.06PSN-0.61PMN-0.33PT.

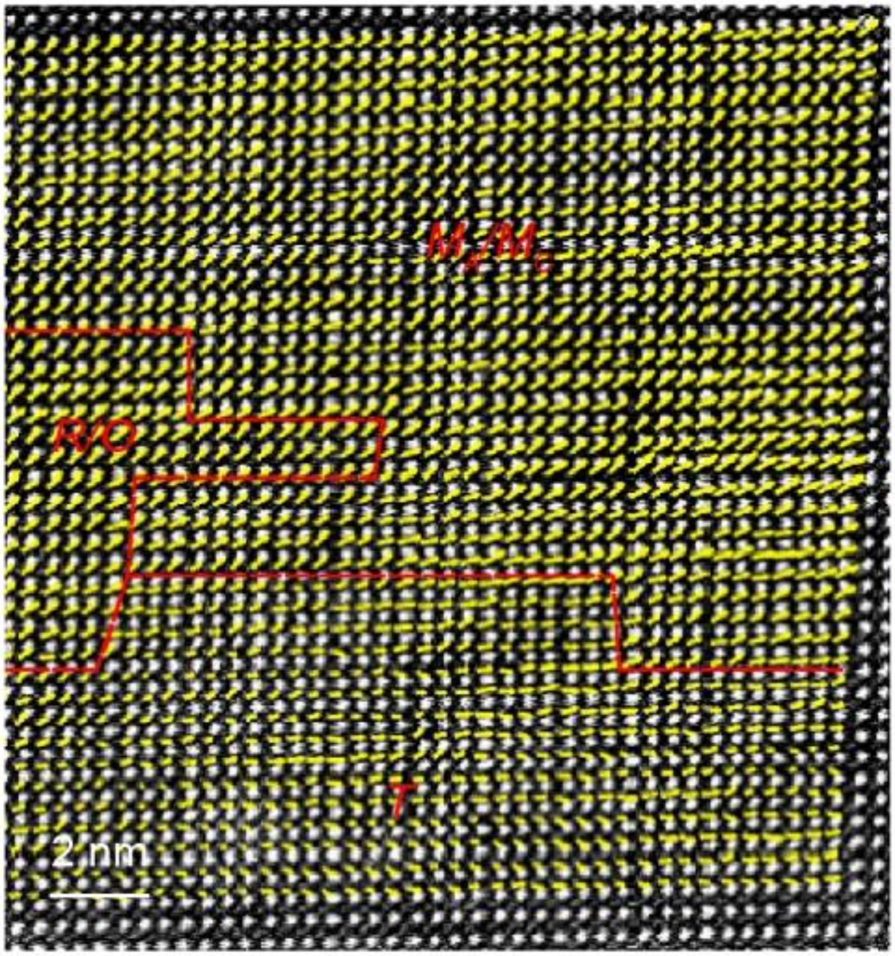

Figure S12. HAADF-STEM image of $[001]_C$ oriented 0.67PMN-0.33PT single crystal, the P_S directions are given for each unit-cell column and the possible phase structure have been labeled.

Note 9. Phase structure and lattice parameters determined by high-resolution XRD.

High-resolution XRD pattern of 0.67PMN-0.33PT is shown in Figure S13, which demonstrates multiphase coexistence: monoclinic M_A (space group *Cm*), monoclinic M_C (space group *Pm*) and tetragonal (space group *P4mm*) phases, consisting with the STEM results (Figure S8). Details phase constitute of 0.06PSN-0.61PMN-0.33PT and 0.67PMN-0.33PT are listed in Table S2. The ternary system exhibits a larger lattice parameter than the binary system due to the larger ionic radius of Sc^{3+} (0.745 Å) than Ti^{4+} (0.605 Å) and Nb^{5+} (0.640 Å). Moreover, the 0.06PSN-0.61PMN-0.33PT contains more tetragonal component than 0.67PMN-0.33PT.

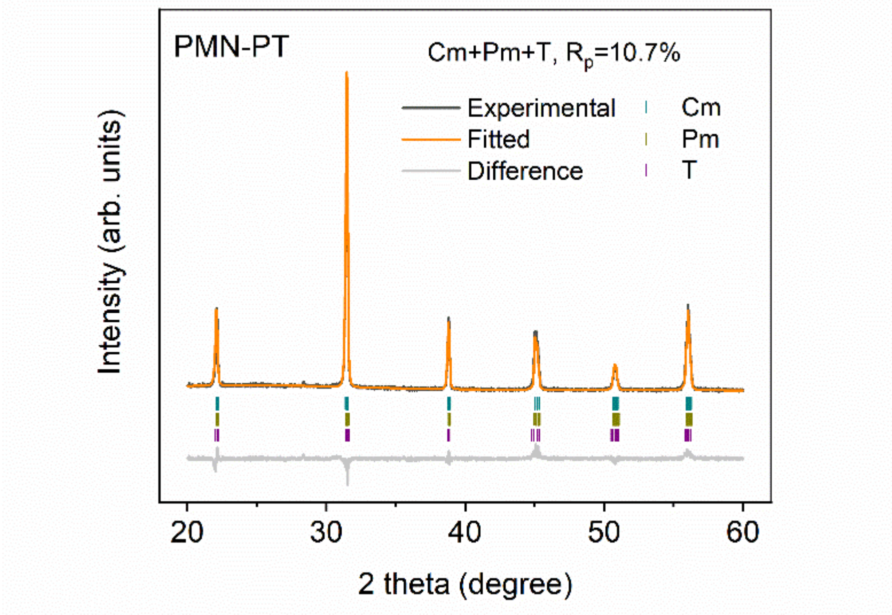

Figure S13. High-resolution XRD pattern and Rietveld refinement of PMN-0.33PT.

Table S2. Phase constitute and lattice parameters for the two crystals basis by high resolution XRD.

**Phase**	**Space Group**	**0.06PSN-0.61PMN-0.33PT**				**0.67PMN-P0.33T**			
		a (Å)	*b* (Å)	*c* (Å)	volume fraction	*a* (Å)	*b* (Å)	*c* (Å)	volume fraction
M_A	*Cm*	5.715	5.704	4.029	43.2%	5.700	5.681	4.010	55.8%
M_C	*Pm*	4.021	4.010	4.032	22.3%	4.017	4.005	4.029	30.5%
T	*P4mm*	4.011	4.011	4.051	34.5%	4.015	4.015	4.044	13.7%

Note 10. Electric field- and temperature-dependent structural evolutions

In Figure S14 we compare the dielectric permittivity *vs.* temperature curves between poled and unpoled 0.06PSN-0.61PMN-0.33PT samples. It is well accepted that during poling process, PNRs transformed into macro ferroelectric domains[8-11], thus after poling the sample has macro ferroelectric domains at room temperature. The dielectric anomaly at $T_{F\text{-}F}$=85 °C of the poled samples corresponds to the temperature-driven ferroelectric to ferroelectric phase transition, along with the partial depoling of the $[001]_C$ poled domain structures. For the unpoled sample, however, the PNR state is always maintained, and no apparent phase structure change occurs below T_m (for details please see the data

shown in Figure S16c), thus the dielectric anomaly corresponding to the ferroelectric-ferroelectric phase transition is not detectable. Other relaxor ferroelectrics also demonstrate similar dielectric behavior[12].

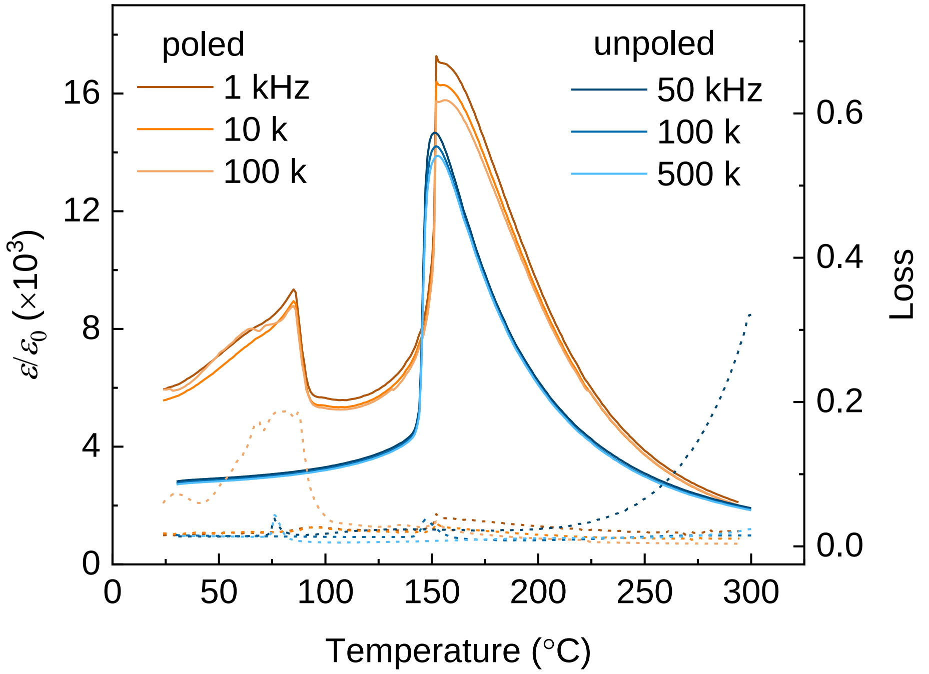

Figure S14. Comparison of dielectric permittivity *vs.* temperature curves of poled and unpoled 0.06PSN-0.61PMN-0.33PT single crystals.

For $[001]_C$ oriented crystal, power XRD characterizations only give information of (00*m*) (*m*=1, 2, 3…) Bragg peaks, thus it is difficult to determine the phase component and lattice parameters using a single crystal sample. Alternatively, we employed polycrystalline ceramics with the same composition as crystal for a detailed temperature-dependent structural study. The main difference between single crystal and ceramics arises from extrinsic factors such as grain boundary, while XRD patterns generally reflect the distortion of the lattice structure, which is an intrinsic factor. Although there may exist a slight difference in structures between single crystal and ceramics, the influence of electric field and temperature should be dominated factor for intrinsic structures here. Thus we believe the results based on ceramics can provide us with quotable information.

Figure S15 demonstrates the XRD patterns of the poled and unpoled samples. At 2θ~45°, the high-angle peak is strengthened after poling, indicating that the domains switch to directions close to the *E*-filed. The narrower diffraction peaks in the poled sample demonstrate more uniform domain structure.

The phase concentrations and lattice parameters for each phase are listed in Table S3. After poling, the component of M_C phase and tetragonal (T) phase increase while the M_A phase decreases.

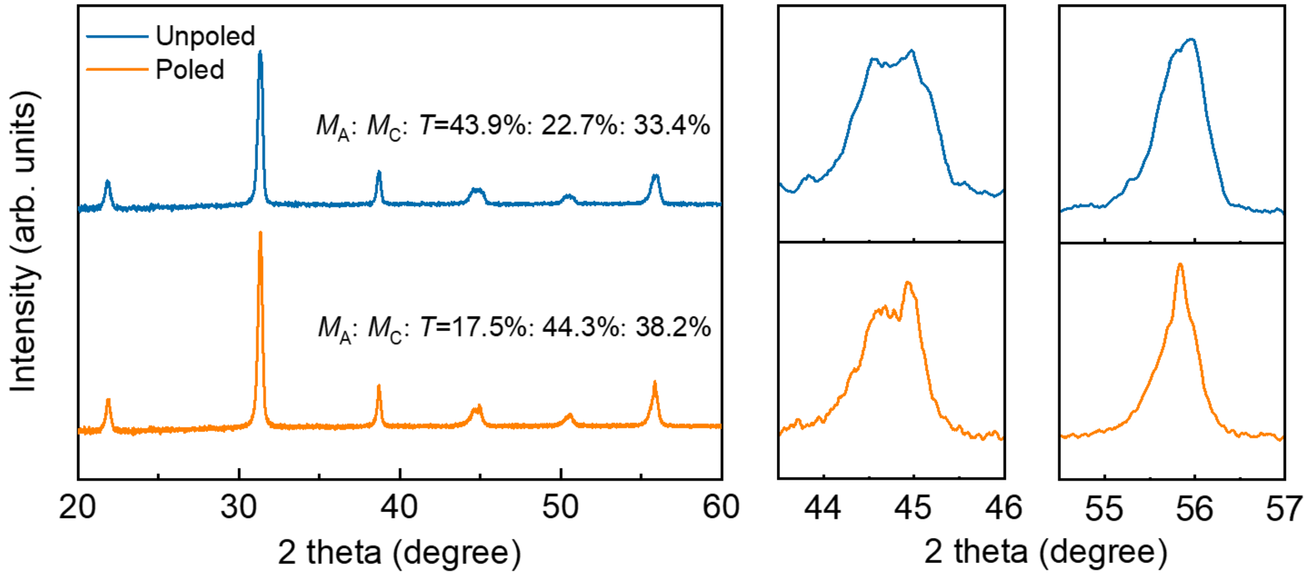

Figure S15. XRD patterns for the poled and unpoeld samples.

Table S3. Phase component and lattice parameters for the poled and unpoeld samples.

**PSN-PMN-0.32PT**	**Unpoled**				**Poled**			
**Lattice parameters**	*a* (Å)	*b* (Å)	*c* (Å)	volume fraction	*a* (Å)	*b* (Å)	*c* (Å)	volume fraction
$\mathbf{M_A}$	5.732	5.687	4.009	43.9%	5.719	5.667	4.000	17.5%
$\mathbf{M_C}$	4.065	3.973	4.032	22.7%	4.037	3.993	4.054	44.3%
**T**	4.001	4.001	4.053	33.4%	4.018	4.018	4.046	38.2%

The XRD patterns as a function of temperature for unpoled and poled samples are shown in Figures S16a and S16b, respectively. The phase fractions are also determined according to our refinements, as shown in Figures S16c and S16d, respectively. With the increase of temperature, M_C phase fraction gradually increases. For the poled samples, the phase fraction changes obviously around T_{F-F}, corresponding well with remarkable dielectric anomaly in Figure S14. The cubic phase begins to appear at around 140 ^oC, and becomes the dominant phase as the temperature increases.

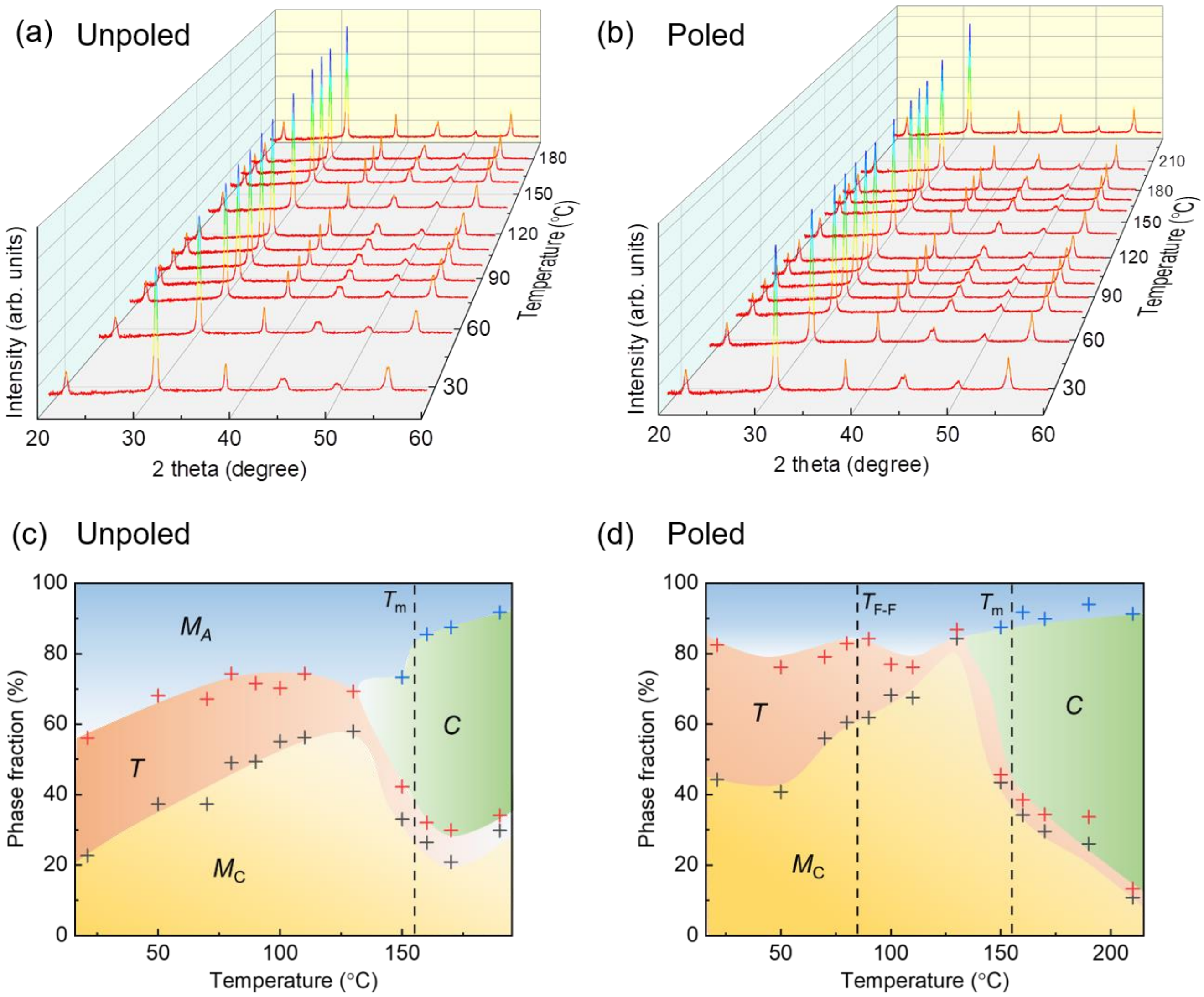

Figure S16. Temperature dependent XRD patterns and calculated phase fractions for (**a**) and (**c**) unpoled and (**b**) and (**d**) poled samples.

Then we discuss the variations of piezoelectric activity with temperature from the aspects of phase structure changes. The total piezoelectric response is the integral effect from all ferroelectric phases: T, M_A and M_C. The tetragonal phase is associated with low d_{33} along $[001]_C$[13,14], and high d_{33} in the 0.06PSN-0.61PMN-0.33PT origins mainly from M_A and M_C phases where P_S rotation contributes greatly. The total volume fraction of M_A+M_C increases with temperature (Figure S17a). In addition, the lattice distortion strengthens with temperature, as demonstrated by the increased β (the angle between lattice a and c) value (Figure S17b). The larger β corresponds to a stronger deviation of P_S vector from $[001]_C$, which could be more sensitive to the external electric field and facilitates piezoelectricity. The

enhanced monoclinic phase fraction, the increased lattice distortion, together with the softened crystal lattice at high temperature give a monotonously enhanced d_{33} with temperature (Figure S4).

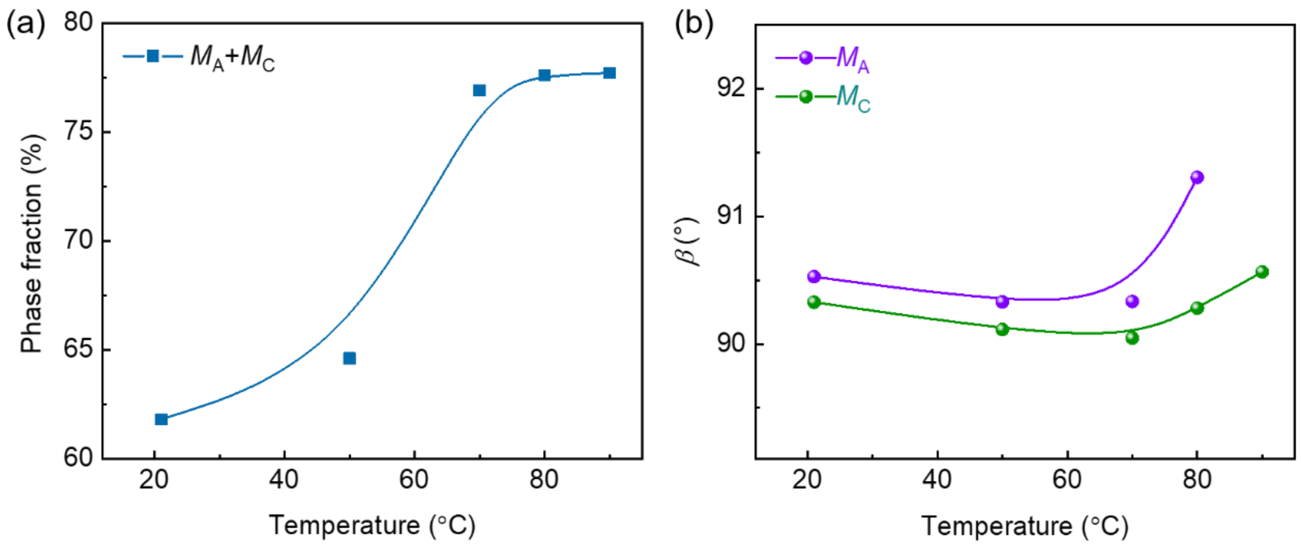

Figure S17. Temperature dependent (a) monoclinic phase fraction and (b) β of M_A and M_C.

Note 11. Phase-field simulations.

In the simulations, we doped tetragonal PMN-0.42PT PNRs into high piezoelectric PMN-0.30PT matrix to theoretically reproduce the situations in PSN-PMN-PT system. Here we chose PMN-0.3PT with pure rhombohedral phase as the parent matrix for simplicity, although differs slightly from the experimental composition, the simulations can still provide guidelines that greatly help to understand the experimental results. The ferroelectrics ceramic of PMN-PT is discretized at grid size is $128\Delta x \times 128\Delta x \times 16\Delta x$, with stress-free boundary conditions, Δx is set to 1 nm. For PMN-0.3PT and PMN-0.42PT, the material coefficients and shown in Table S3.[15] Figure S18a shows the calculated piezoelectric constant d_{33} of PMN-PT with and without tetragonal component doping. d_{33} of pure PMN-PT is 729 pC/N, enhancing to 1641 pC/N by introducing tetragonal component. Besides, the strong piezoelectric in the doped PMN-PT is highly homogenous (Figure S18c). The *P*-*E* hysteresis loops of these two systems were also calculated (Figure S18b), from which we observe a significant enhancement magnitude of E_C due to the appropriate doping of tetragonal component.

Table S4. Materials coefficients for PMN-0.3PT and PMN-0.42PT used in this work.

Coefficient	PMN-0.3PT	PMN-0.42PT
α_1 (10^5 C^{-2} m^2 N)	2.295×T-935.9	2.583×T-1204
α_{11}(10^5 C^{-4} m^6 N)	-0.3775×T+457.7	-0.3775×T+304.2
α_{12}(10^7 C^{-4} m^6 N)	6.075	10.85
α_{111}(10^9 C^{-6} m^{10} N)	2.57	2.57
α_{112}(10^9 C^{-6} m^{10} N)	6.95	6.95
α_{123}(10^9 C^{-6} m^{10} N)	13.13	13.13
S_{11}(10^{-12} m^2/N)	52	9.43
S_{12}(10^{-12} m^2/N)	-18.9	-1.68
S_{44}(10^{-12} m^2/N)	14	35.09
Q_{11}(m^4/C^2)	0.084	0.084
Q_{12}(m^4/C^2)	-0.025	-0.025
Q_{44}(m^4/C^2)	0.035	0.035

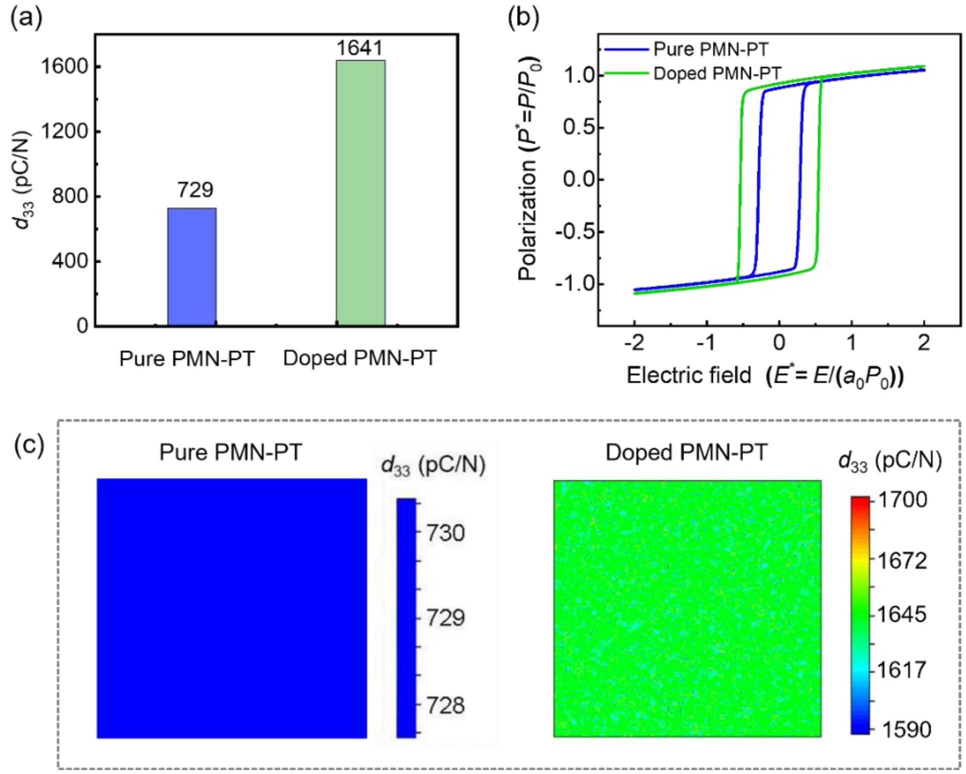

Figure S18. Phase-field simulations of (**a**) Piezoelectric constants d_{33}, (**b**) *P-E* loops and (**c**) distribution of d_{33} for pure and doped PMN-PT.

Note 12. Highly disordered structure and random fields

The relaxor-$PbTiO_3$ ferroelectrics are charge-disordered relaxors. The inherent charge disorder is due to the random distribution of various B-site ions such as Mg^{2+}, Nb^{5+} and Ti^{4+}. It offers uncorrelated and quenched random electric fields at the sites of the ferroelectric-active ions. These random fields favor the formation of PNRs. The dynamic PNRs begin to appear at Burn temperature T_B. The interaction between PNRs and quenched random electric fields results in a local mesoscale phase transition at T*, below which the static PNR appears, coexisting with dynamic ones. On further cooling, the slowdown of PNR dynamics result in a remarkable dielectric relaxation. Finally, below freezing temperature, T_{VF}, the PNR dynamics ultimately slow down into a totally static glassy-like state[6,16-18].

The introduction of a small amount of trivalent Sc^{3+} ions make the B-site ions more disordered than the PMN-PT system, thus a stronger random field formed. As a result, the dynamic PNRs appear at a higher temperature, as verified by the experimental results that the T_B of 0.06PSN-0.61PMN-0.33PT is 40 ^oC higher than the 0.67PMN-0.33PT (Figure 3a). The highly disordered state and strong random field can be further confirmed by the higher diffused factor γ (Figure 3b), the lower freezing temperature T_{VF} (Figure 3f), the larger T_m shift (Figure 3c and Figure S10), the smaller domain size and the shorter correlation length ξ (Figure S8).

It should be noted that to obtain highly disordered state, the amount of doped Sc^{3+} should be small. If the system contains a large amount of PSN, the chemically ordered regions with regular Sc^{3+}-Nb^{5+} ordering could be easily established, and a weak but homogeneous local field is formed at the chemically ordered regions, giving raise to preferential local order ferroelectric domain[16].

Note 13. Domain switching

PFM technique was employed to explore the domain dynamic behavior. An area of 6×6 μm^2 is pre-poled by a tip voltage of -20 V to form an upward region. Then nanoscale domains in this region were reversed downward with different positive tip voltages and pulse durations. Figure S19 shows the out-of-plane phase image of the domains. For the 0.67PMN-0.33PT single crystal, only one nucleated domain grows till to complete a domain switching. The domain diameter increases with the increase of both tip voltage and pulse duration, and the domain growth is the predominant effect. These results are consistent with previous results on some other ferroelectrics crystals and films[19-21]. However, for 0.06PSN-0.61PMN-0.33PT, the domain switching is expedited by an increased amount of nucleation

sites. This is corroborated by the irregular shape of the switched area arising from many individually nucleated domains, which may coalesce during the growth. The multi-site domain nucleation phenomena has been observed in ferroelectric Pb(Zr,Ti)O_3 film, which contains a large amount of defect pinning centers[22,23]. As such, it is reasonably deduced that the domain growth in 0.06PSN-0.61PMN-0.33PT is severely inhibited by the high concentration of pinning centers, *e.g.*, tetragonal nanoclusters, and domain nucleation is the dominant effect for domain dynamics.

In addition, domain nucleation in the 0.06PSN-0.61PMN-0.33PT is also more difficult than in the 0.67PMN-0.33PT. The domains in the yellow rectangle are fabricated at different pulse durations with a fixed tip voltage of 7 V. As can be seen, domain nucleation occurs with a smaller pulse duration in the binary system than in the ternary one. On the other hand, with a fixed pulse duration of 3 s, the domain nucleation also happens at a lower tip voltage (domains in the red rectangle) for the 0.67PMN-0.33PT. These results further support the observed higher E_C in ternary crystals.

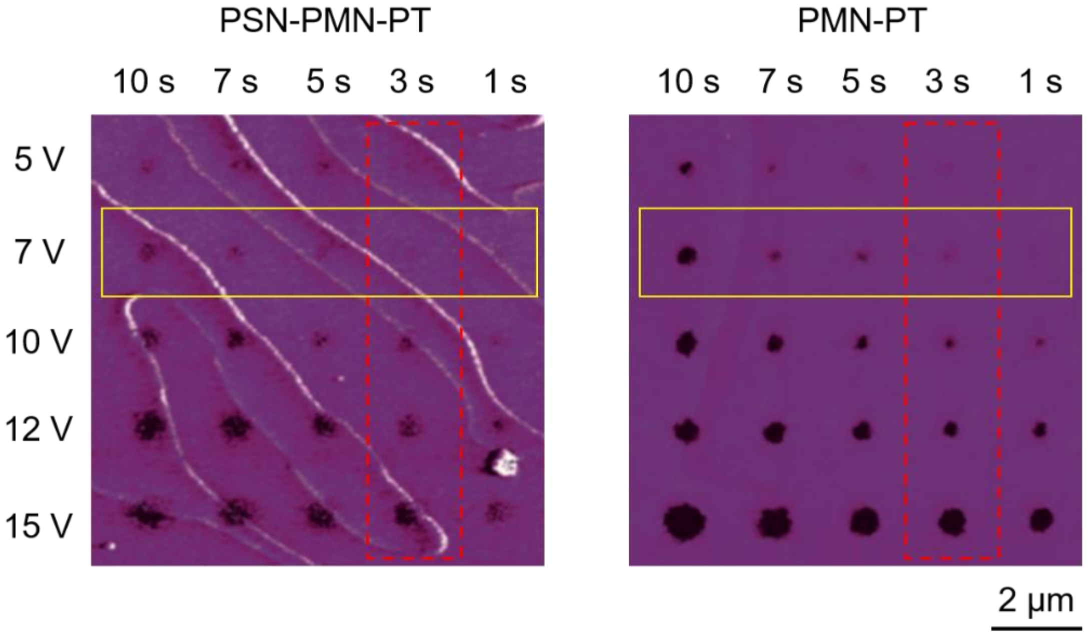

Figure S19. Domain switching behavior under different tip voltage and pulse duration.

We then consider the dynamics of domain switching. The activation electric field E_A required for domain switch is determined by Merz's law. Figure S2a shows the *P-E* hysteresis loops measured at different frequencies *f*. The frequency dependent E_C can be depicted by Merz's law[24-26]:

$$\tau \propto 1/f \propto \exp(E_A/E_C), \tag{S2}$$

where τ is the switching time. Figure S20 gives the fitting results of the experimental data. A lager value of E_A=138 kV/cm was derived for 0.06PSN-0.61PMN-0.33PT, in comparison of 23 kV/cm for 0.67PMN-0.33PT. The higher E_A of ternary system means a more difficult domain reverse process, further verifying the proposed deep potential barrier ΔG (Figure 5).

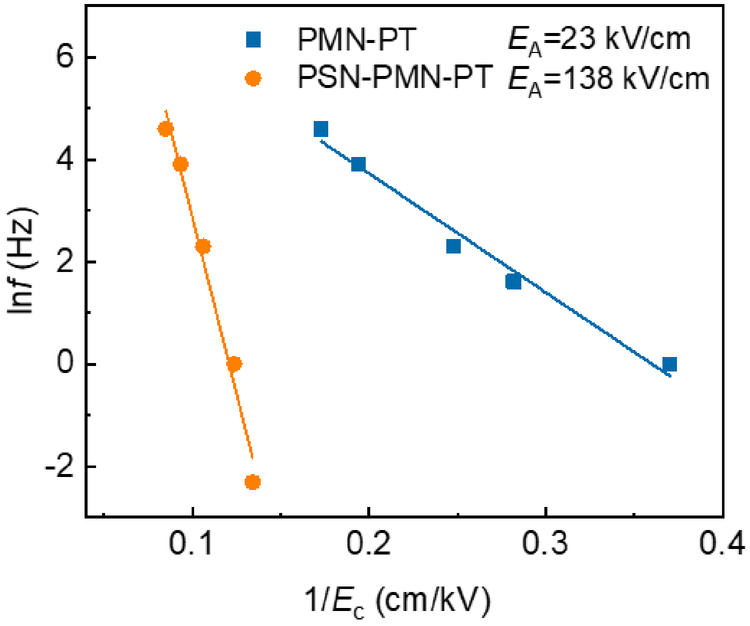

Figure S20. 1/E_C *vs.* ln$f$ of 0.06PSN-0.61PMN-0.33PT and 0.67PMN-0.33PT crystals. The solid lines are the fitting results of Merz's law.

Note 14. Longitudinal and shear piezoelectric response.

Longitudinal piezoelectric coefficient d_{33} and shear coefficient d_{15} correspond to different kinds of lattice deformation in response to electric stimulate. For the 33-mode, the measured electric filed is applied along $[001]_C$, leading to a longitudinal lattice deformation through electromechanical coupling (Figure S21a), whereas for the 15-mode, shear lattice deformation occurs under external field along $[100]_C$ (Figure S21b). Figure S22 demonstrates the piezoelectric lattice deformation of d_{33} and d_{15} modes for various engineered domain configurations. Generally, the 4R and 4O domain structures exhibit large longitudinal piezoelectric coefficient d_{33} while the 1T domain structure shows high shear response d_{15} for the easier polarization rotation.[27]

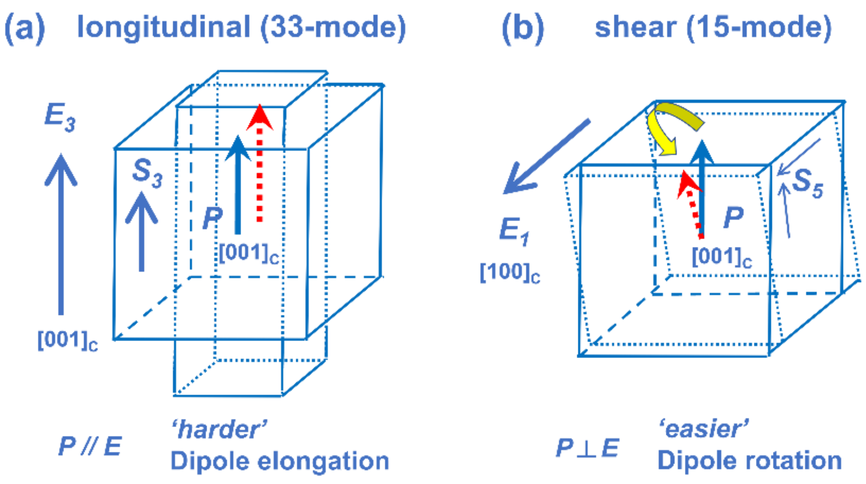

Figure S21. Diagrams of (**a**) longitudinal and (**b**) shear lattice deformation for $[001]_C$ poled samples. The vectors *P* demonstrate total polarization along $[001]_C$ rather than the spontaneous polarization.

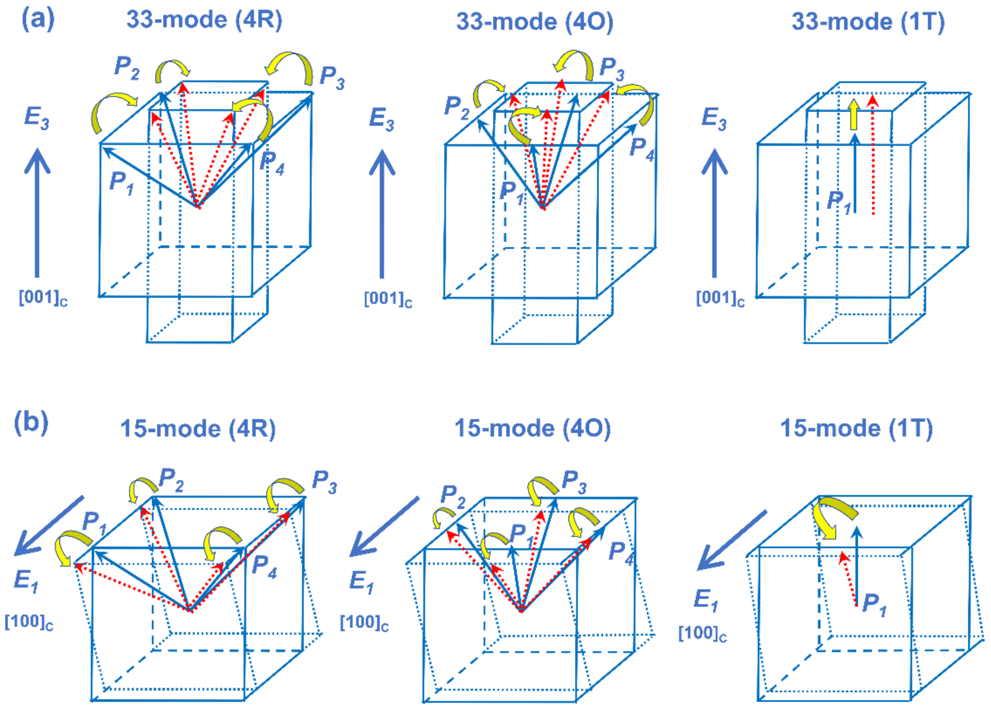

Figure S22. Piezoelectric deformation of crystal lattice with various engineered domain configurations: (**a**) d_{33} and (**b**) d_{15} mode of 4R ($[001]_C$ poled rhombohedral phase), 4O ($[001]_C$ poled orthorhombic phase), and 1T ($[001]_C$ poled tetragonal phase). The vectors *P* demonstrate spontaneous polarizations for each domain structures.

Note 15. Property comparison among various PSN-PMN-PT crystals

In Table S5 we listed the main performance of our 0.06PSN-0.61PMN-0.33PT single crystal in comparison with previous work.[28,29] One can clearly see that our work demonstrates superior properties: d_{33}~2630 pC/N is 2 times higher than those in Work 1 (~1200 pC/N) and Work 2 (1260-1550 pC/N), and meanwhile E_C~8.2 kV/cm is much larger than those in Works 1 and 2 (4-6 kV/cm). In addition, we observed that the electromechanical coupling factor (k_{33}~0.9) and dielectric constant (ε_{33}~5950) of our crystals are also superior. The PSN-PMN-PT crystals in Works 1 and 2 demonstrate relatively low d_{33} and moderate E_C, comparable to the PIN-PMN-PT:Mn and PSN-PT systems, at an average level among all the data shown in Figure 1a. Similar to other works, the enhancement of E_C in Works 1 and 2 is at the expense of piezoelectricity d_{33}. Alternatively, our crystal achieves simultaneous ultrahigh piezoelectricity and extremely large coercive field, far beyond the boundary (see the red dash line in Figure 1a) of other systems, getting access the previously "no data" region.

Table S5. Comparison of the main performance for PSN-PMN-PT system.

	This work	Work 1 (ref. 28)	Work 2 (ref. 29)
Composition	0.06PSN-0.61PMN-0.33PT	0.05PSN-0.63PMN-0.32PT	*y*PSN–*z*0.63PMN-xPT<br>*x*=0.12-0.13, *z*=0.52-0.48;<br>*x*=0.26-28, *z*=048-0.435;<br>*x*=0.3675-0.3975, *z*=0.51-0.47
Phase structure	**MPB**	R	R, MPB, T
d_{33} (pC/N)	**2630**	1200	1260-1550
E_C (kV/cm)	**8.2**	4-6[b]	4-6
ε_{33} (ε_0)	**5950**	3500	1700-2000
k_{33}	**0.9**	-	~ 0.75
γ	**1.96**	1.73[a]	1.65-1.82[a]
$T_{F\text{-}F}$ (^oC)	**85**	70	120-180
T_C (^oC)	**152**	162	200-240
Domain size (nm)	**~200**	-	~ 1000

[a] The diffuseness factor γ, which was originally lacking in this manuscript, has been calculated from the dielectric permittivity vs. temperature curves, as shown in Figure S23; [b] The E_C value is not provided in ref. 28. Generally, the ferroelectrics is poled by a field exceeding $2E_C$. Considering that the poling electric field is 10 kV/cm, E_C is estimated to be 4-6 kV/cm.

The diffuseness factor γ for the sample in Work 1 is 1.72, and 1.65-1.82 in Work 2 (Table S5 and Figure S23), much lower than the value in our work (γ=1.94). In addition, the crystals in Work 2 demonstrate a much larger domain size. Both small γ value and large domain size verify less dispersed micro polar structure, therefore gives low piezoelectric properties.

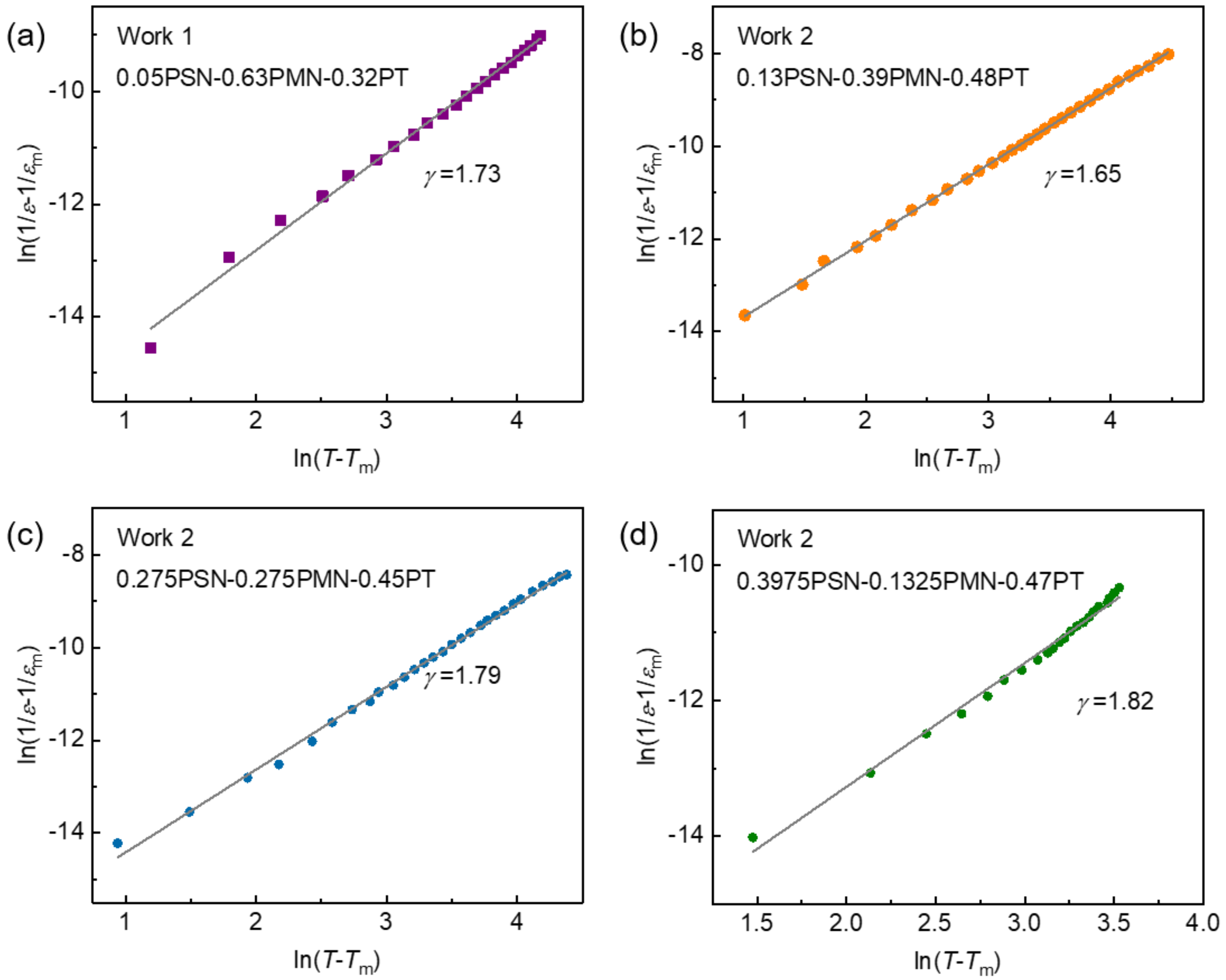

Figure S23. Diffuse factor γ for the samples studied in Works 1 and 2.

Note 16. Domain wall mobility and lattice deformation under external stimuli

Rayleigh analysis was performed to estimate the domain wall mobility under the external E field.[30,31]

$$\varepsilon_{33}(E_0) = \varepsilon_{\mathrm{rev}} + \alpha \cdot E_0 \tag{2}$$

$$P(E) = \varepsilon_0[(\varepsilon_{\mathrm{rev}} + \alpha \cdot E_0)E \pm \alpha(E_0^2 - E^2)/2]. \tag{3}$$

Where $\varepsilon_{33}(E_0)$ is the dielectric permittivity under an AC electric field with amplitude E_0, αE_0 describes irreversible dielectric contributions from domain wall motions, and α is the Rayleigh parameter. $\varepsilon_{\mathrm{rev}}$ is the reversible dielectric response, which mainly comes from lattice deformation. Figure S24 shows the Rayleigh behavior of $[001]_C$ poled 0.06PSN-0.61PMN-0.33PT and 0.67PMN-0.33PT crystals. α of PSN-PMN-0.33PT is 3185 cm/kV, 3 times higher than that for 0.67PMN-0.33PT (902 cm/kV), corresponding to a high level of domain wall mobility. $\varepsilon_{\mathrm{rev}}$ of 0.06PSN-0.61PMN-0.33PT is 7628, 60% higher than that of PMN-PT (4596), demonstrating an easier lattice deformation under external stimuli.

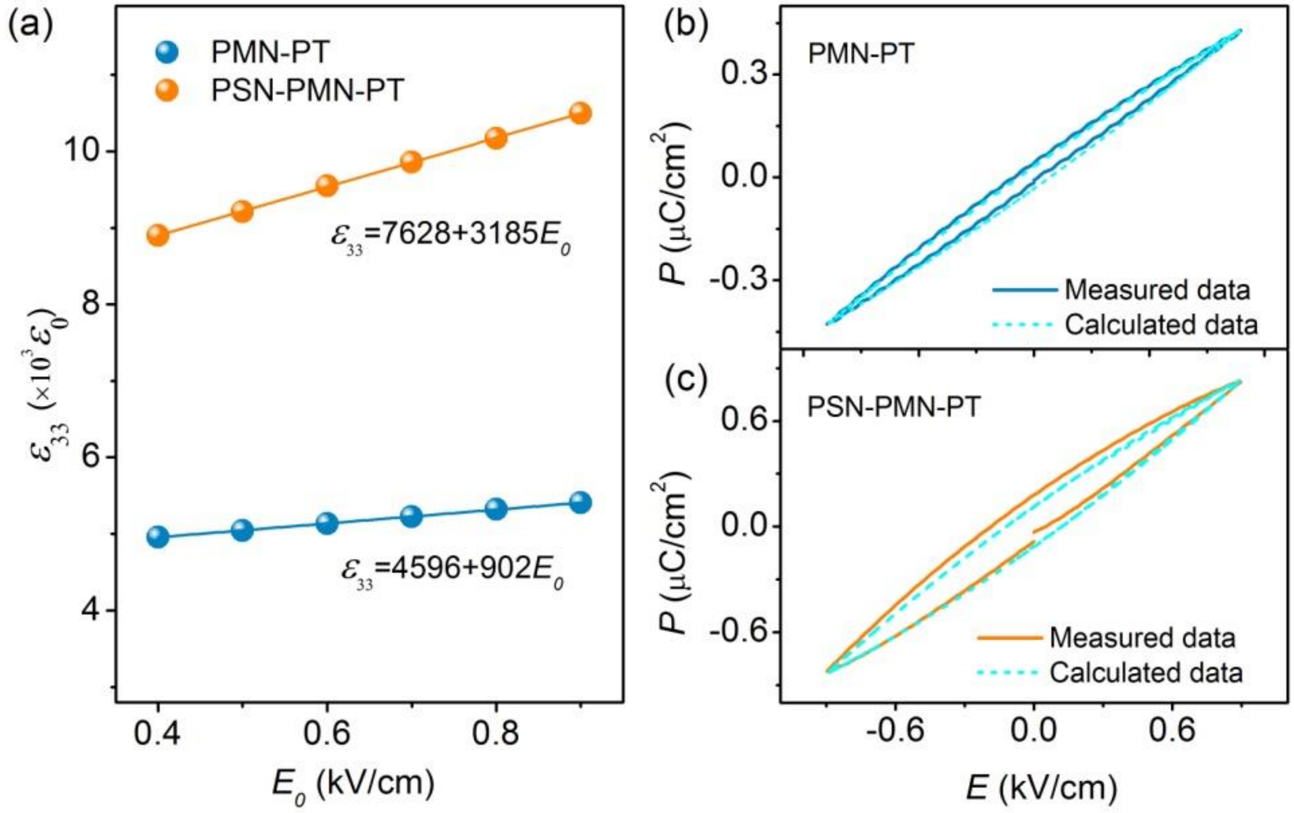

Figure S24. Rayleigh analysis of 0.67PMN-0.33PT and 0.06PSN-0.61PMN-0.33PT single crystals. (**a**) ε_{33} as a function of E_0 and the linear fitting results. (**b**) Comparison between the measured and calculated *P*-*E* hysteresis loops of 0.67PMN-0.33PT crystal. (**c**) Comparison between the measured and calculated *P*-*E* hysteresis loops of 0.06PSN-0.61PMN-0.33PT crystal.

Rayleigh analysis as a function of temperature was also carried out (Figure S25a) and the reversible dielectric constant $\varepsilon_{\mathrm{rev}}$ and Rayleigh parameter α as a function of temperature are summarize in Figure S25b. $\varepsilon_{\mathrm{rev}}$ dramatically enhances as the phase transition temperature $T_{\text{F-F}}$ is approached. The total incensement is around 2 times, from 7628 at room temperature to 15215 around $T_{\text{F-F}}$. The enhancement

of α is much more distinct in comparison with ε_{rev}, which grows exponentially by 7 times from 3185 cm/kV to 24127 cm/kV, corresponding to the greatly improved domain wall motions.

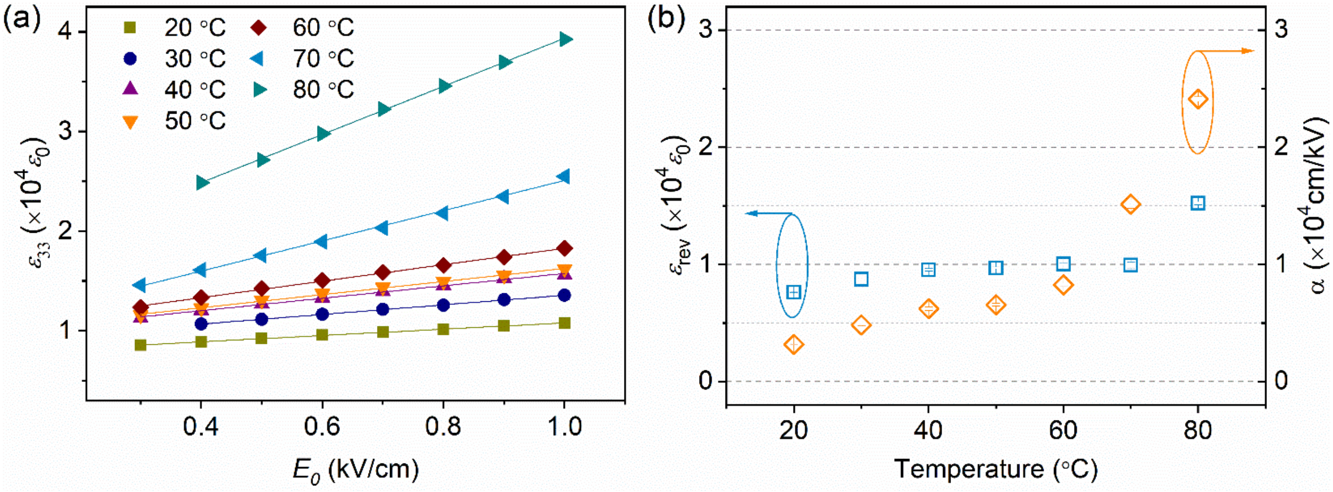

Figure S25. Temperature dependent Rayleigh behavior of 0.06PSN-0.61PMN-0.33PT single crystal. (**a**) ε_{33} and (**b**), α and ε_{rev} at various temperatures.

Supplementary references:

1. Rajasekaran SV, Achary SN, Patwe SJ, Jayavel R, Mangamma G & Tyagi AK. Phase transformation in relaxor-ferroelectric single crystal $0.58Pb(Sc_{1/2}Nb_{1/2})O_3$-$0.42PbTiO_3$. *J Mater Res* **29**, 1054 (2014).

2. Singh AK, Pandey D. Evidence for M_B and M_C phases in the morphotropic phase boundary region of $(1-x)Pb(Mg_{1/3}Nb_{2/3})O_3$-$xPbTiO_3$: A Rietveld study. *Phys Rev B* **67**, 064102 (2003).

3. Scott J F. Models for the frequency dependence of coercive field and the size dependence of remanent polarization in ferroelectric thin films. *Integr Ferroelectr* **12**: 71-81 (1996).

4. Nomura Y, Tachi T, Kawae T, et al. Temperature dependence of ferroelectric properties and the activation energy of polarization reversal in (Pr, Mn)-codoped $BiFeO_3$ thin films. *Phys Status Solidi B* **252**: 833-838 (2015).

5. Tai CW, Baba-Kishi KZ. Relationship between dielectric properties and structural long-range order in $xPb(In_{1/2}Nb_{1/2})O_3$-$(1-x)Pb(Mg_{1/3}Nb_{2/3})O_3$ relaxor ceramics. *Acta Mater* 54, 5631-5640 (2006).

6. Shvartsman VV, Kleemann W, Łukasiewicz T, Dec J. Nanopolar structure in $Sr_xBa_{1-x}Nb_2O_6$ single crystals tuned by Sr/Ba ratio and investigated by piezoelectric force microscopy. *Phys Rev B* 77, 054105 (2008).

7. Shvartsman VV, Dkhil B, Kholkin AL. Mesoscale domains and nature of the relaxor state by piezoresponse force microscopy. *Annu Rev Mater Res* 43, 423-449 (2013).

8. Li J, Li J, Qin S, et al. Effects of long-and short-range ferroelectric order on the electrocaloric effect in relaxor ferroelectric ceramics. *Phys Rev Appl* **11**: 044032 (2019).

9. Zuo R, Li F, Fu J, et al. Electric field forced c-axis oriented growth of polar nanoregions and rapid switching of tetragonal domains in BNT-PT-PMN ternary system. *J Eur Ceram Soc* **36**: 515-525 (2016).

10. Zhou X, Jiang C, Luo H, et al. Enhanced piezoresponse and electric field induced relaxor-ferroelectric phase transition in NBT-0.06BT ceramic prepared from hydrothermally synthesized nanoparticles. *Ceram Int* **42**(16): 18631-18640 (2016).

11. Zaman A, Hussain A, Malik R A, et al. Dielectric and electromechanical properties of LiNbO3-modified (BiNa)TiO_3–(BaCa)TiO_3 lead-free piezoceramics. *J Phys D: Appl Phys* **49**(17): 175301 (2016).

12. Lin D, Li Z, Zhang S, et al. Electric-field and temperature induced phase transitions in Pb($Mg_{1/3}$ $Nb_{2/3}$)O_3–0.3PbTiO_3 single crystals. *J Appl Phys* **108**(3): 034112 (2010).

13. Wang L, Zhai Y, Zheng L, et al. Intrinsic piezoelectricity in (K, Na) NbO_3-based lead-free single crystal: Piezoelectric anisotropy and its evolution with temperature. *Appl Phys Lett* **117**: 052904 (2020).

14. Zheng L, Jing Y, Lu X, et al. Temperature dependent piezoelectric anisotropy in tetragonal 0.63Pb($Mg_{1/3}Nb_{2/3}$)O_3-0.37PbTiO_3 single crystal. *Appl Phys Lett* **113**(10): 102903 (2018).

15. Liu D, Zhao R, Jafri HM, et al. Phase-field simulations of surface charge-induced polarization switching. *Appl Phys Lett* **114**, 112903 (2019)

16. Tinte S, Burton B P, Cockayne E, et al. Origin of the relaxor state in Pb($B_xB_{1-x'}$)O_3 perovskites. *Phys Rev Lett* **97**(13): 137601 (2006).

17. Westphal V, Kleemann W, Glinchuk M D. Diffuse phase transitions and random-field-induced domain states of the "relaxor" ferroelectric Pb($Mg_{1/3}Nb_{2/3}$)O_3. *Phys Rev Lett* **68**(6): 847 (1992).

18. `Kleemann W. Relaxor ferroelectrics: Cluster glass ground state via random fields and random bonds. Phys Status Solidi B **251**(10):1993–2002 (2014).

19. Rose L Y, Halliwill K D, Adams C J, et al. Mutational signatures in tumours induced by high and low energy radiation in Trp53 deficient mice. *Nat Commun* **11**(1): 1-15 (2020).

20. Bakaul S R, Kim J, Hong S, et al. Ferroelectric Domain Wall Motion in Freestanding Single-Crystal Complex Oxide Thin Film. *Adv Mater* **32**(4): 1907036 (2020).

21. He W, Li Q, Sun Y, et al. Investigation of piezoelectric property and nanodomain structures for PIN–PZ–PMN–PT single crystals as a function of crystallographic orientation and temperature. *J Mater Chem C* **5**(9): 2459-2465 (2017).

22. McGilly L J, Sandu C S, Feigl L, et al. Nanoscale defect engineering and the resulting effects on domain wall dynamics in ferroelectric thin films. *Adv Funct Mater* **27**(15): 1605196 (2017).

23. Jakes P, Erdem E, Eichel R A, et al. Position of defects with respect to domain walls in Fe^{3+}-doped $Pb[Zr_{0.52}Ti_{0.48}]O_3$ piezoelectric ceramics. *Appl Phy Lett* **98**, 072907 (2011).

24. Jiang A Q, Chen Z H, Hui W Y, et al. Subpicosecond domain switching in discrete regions of Pb $(Zr_{0.35}Ti_{0.65})O_3$ thick films. *Adv Funct Mater* **22**(10): 2148-2153 (2012).

25. Schultheiß J, Liu L, Kungl H, et al. Revealing the sequence of switching mechanisms in polycrystalline ferroelectric/ferroelastic materials. *Acta Mater* **157**: 355-363 (2018).

26. Scott J F. Switching of ferroelectrics without domains. *Adv Mater* **22**, 5315-5317 (2010).

27. Zhang S, Li F. High performance ferroelectric relaxor-$PbTiO_3$ single crystals: Status and perspective. *J Appl Phys* **111**, 2-27 (2012).

28. Guo Y, Xu H, Luo H, et al. Growth and electrical properties of $Pb(Sc_{1/2}Nb_{1/2})O_3$–Pb $(Mg_{1/3}Nb_{2/3})O_3$–$PbTiO_3$ ternary single crystals by a modified Bridgman technique. *J Cryst Growth* **226**(1): 111-116 (2001).

29. Wang Z, He C, Qiao H, et al. In situ di-, piezo-, ferroelectric properties and domain configurations of $Pb(Sc_{1/2}Nb_{1/2})O_3$–$Pb(Mg_{1/3}Nb_{2/3})O_3$–$PbTiO_3$ ferroelectric crystals. *Cryst Growth Des* **18**(1): 145-151 (2018).

30. Griggio F, Jesse S, Kumar A, et al. Substrate clamping effects on irreversible domain wall dynamics in lead zirconate titanate thin films. *Phys Rev Lett* **108**, 157604 (2012).

31. Garcia J E, Perez R, Ochoa DA, et al. Evaluation of domain wall motion in lead zirconate titanate ceramics by nonlinear response measurements. *J Appl Phys* **10**, 6445 (2008).
